# Adenosine receptor ligation tips the uveitogenic Th1 and Th17 balance towards the latter in experimental autoimmune uveitis-induced mouse

**DOI:** 10.1016/j.crimmu.2021.07.001

**Published:** 2021-07-26

**Authors:** Deming Sun, Minhee Ko, Hui Shao, Henry J. Kaplan

**Affiliations:** a Doheny Eye Institute and Department of Ophthalmology, David Geffen School of Medicine at UCLA, Los Angeles, CA, 90033, United States; b Department of Ophthalmology and Visual Sciences, Kentucky Lions Eye Center, University of Louisville, Louisville, KY, 40202, United States; c Saint Louis University (SLU) Eye Institute, SLU School of Medicine, Saint Louis, MO, 63104, United States

**Keywords:** Adenosine, Adenosine deaminase, Autoimmune response, γδ T cells, Th17

## Abstract

Various pathological conditions are accompanied by release of adenosine triphosphate (ATP) from the intracellular to the extracellular compartment, where it degrades into adenosine and modulates immune responses. Previous studies concluded that both ATP and its degradation product adenosine are important immune-regulatory molecules; ATP acted as a danger signal that promotes immune responses, but adenosine's effect was inhibitory. We show that adenosine receptor ligation plays an important role in balancing Th1 and Th17 pathogenic T cell responses in experimental autoimmune uveitis (EAU). While its effect on Th1 responses is inhibitory, its effect on Th17 responses is enhancing, thereby impacting the balance between Th1 and Th17 responses. Mechanistic studies showed that this effect is mediated via several immune cells, among which γδ T cell activation and dendritic cell differentiation are prominent; adenosine- and γδ-mediated immunoregulation synergistically impact each other's effect. Adenosine receptor ligation augments the activation of γδ T cells, which is an important promoter for Th17 responses and has a strong effect on dendritic cell (DC) differentiation, tipping the balance from generation of DCs that stimulate Th1 responses to those that stimulate Th17 responses. The knowledge acquired in this study should improve our understanding of the immune-regulatory effect of extracellular ATP-adenosine metabolism and improve treatment for autoimmune diseases caused by both Th1- and Th17-type pathogenic T cells.

## Highlights

• Adenosine receptor ligation plays an important role in balancing Th1 and Th17 pathogenic T cell responses in autoimmune diseases.
• The effect of adenosine on Th1 responses is inhibitory and its effect on Th17 responses is enhancing.
• Adenosine- and γδ-mediated immunoregulation synergistically impact each other's effect.

## 1 Introduction

The purinergic system is an evolutionally selected system modulating immune responses (Haskó et al., 2008; Junger, 2011). Under physiological conditions, adenosine triphosphate (ATP) is contained exclusively within cells; however, almost all types of mammalian cells are able to release ATP during tissue damage and inflammation (Junger, 2011). Upon entering the extracellular space, ATP is hydrolyzed into adenosine diphosphate, adenosine-5′-monophosphate, and finally, adenosine in a stepwise manner by ectonucleotidases, including CD73 and CD39 (Fredholm et al., 2011; Haskó et al., 2008; Jacobson and Gao, 2006; Sauer et al., 2012; Yegutkin, 2008). Previous studies have shown that while ATP acts like an endogenously generated Toll-like receptor (TLR) ligand capable of augmenting immune responses (Beigi et al., 2003; Canaday et al., 2002; Hanley et al., 2004; Wilkin et al., 2001), the ATP metabolite adenosine is profoundly anti-inflammatory (Antonioli et al., 2013; Eltzschig and Carmeliet, 2011; Haskó et al., 2009; Naganuma et al., 2006; Ohta and Sitkovsky, 2001; Zarek et al., 2008). An increase in extracellular adenosine reduces the local inflammatory response, while removal of endogenous adenosine aggravates tissue dysfunction elicited by injury (Grenz et al., 2011). Binding of adenosine to its receptors modulates various pathophysiological responses, including immune responses (Fredholm et al., 2011; Haskó et al., 2008; Jacobson and Gao, 2006; Sauer et al., 2012). The discovery of the regulatory effect of adenosine on inflammation and immune responses has led to attempts to treat immune dysfunctions by targeting adenosine receptor (AR) signaling (Haskó et al., 2008; Jacobson and Gao, 2006). Targeting of ARs and adenosine generation has been successful in treating cancer and neurological diseases (Cronstein et al., 1991; Jacobson and Gao, 2006; Ramlackhansingh et al., 2011).

The extrapolation of adenosine as inhibitory was mostly obtained from studies of Th1-type (interferon (IFN)-γ-producing cells) immune responses, since Th17 responses were discovered only recently. Given the available knowledge that both Th1 and Th17 pathogenic T cells contribute to the pathogenesis of autoimmune diseases (Bettelli et al., 2006; Cua et al., 2003; Dong, 2006), determination of whether adenosine has a similar effect on Th1 and Th17 pathogenic T cell responses is important. In this study we show that the effect of AR ligation on Th17 responses is fundamentally different than its effect on Th1 responses; while it inhibits Th1 responses, it enhances Th17 responses. Mechanistic studies showed that the enhancing effect of adenosine on Th17 responses is accomplished via a sum of effects on various other cellular responses important for T cell activation, including αβ T cells, γδ T cells, DCs and regulatory T cells. Adenosine is an important co-stimulating molecule for γδ T cell activation, and augmented γδ T cell activation leads to high Th17 responses (Cui et al., 2009; Liang et al., 2013b, 2016a; Nian et al., 2010). We also show that adenosine exposed DCs showed a greater stimulating effect on γδ T cell activation. Thus, reciprocal interaction between γδ T cells and DCs leads to enhanced Th17 responses. Adenosine and γδ-based treatments should be more successful if the mechanisms by which they affect Th1 and Th17 responses are better understood.

## 2 Materials and methods

### 2.1 Animals and reagents

All animal studies conformed to the Association for Research in Vision and Ophthalmology statement on the use of animals in Ophthalmic and Vision Research. Institutional approval by Institutional Animal Care and Use Committee (IACUC) of Doheny Eye Institute, University of California Los Angeles was obtained, and institutional guidelines regarding animal experimentation were followed.

Female C57BL/6 (B6) and TCR-δ^−/−^ mice on the B6 background were purchased from Jackson Laboratory (Bar Harbor, ME, USA). A2AR^−/-^ mice (Chen et al., 1999) were a gift from Dr. Jiang-Fan Chen (Boston University School of Medicine, Boston, MA, USA). They were housed and maintained in the animal facilities of the University of California Los Angeles. Recombinant murine IL-1β, IL-7, and IL-23 were purchased from R & D Systems (Minneapolis, MN, USA). Fluorescein isothiocyanate (FITC)-, phycoerythrin (PE)-, or allophycocyanin-conjugated antibodies (Abs) against mouse CD4 (GK1.5), αβ T cell receptor (TCR) (H57-597), or γδ TCR (GL3) and their isotype control antibodies were purchased from Biolegend (San Diego, CA, USA). (PE)-conjugated anti-mouse IFN-γ (XMG1.2) and IL-17(TC11-18H10.1) monoclonal antibody was purchased from Santa Cruz Biotechnology (Dallas, TX, USA). The non-selective AR agonist 5′-N-ethylcarboxamidoadenosine (NECA) (Mahamed et al., 2015), selective A2AR agonist 2-p-(2-carboxyethyl) phenethylamino-5′-N-ethylcarboxamidoadenosine (CGS21680), selective A1R agonist (CCPA), A2BR agonist BAY60–6538, A2AR antagonist (SCH 58261) (Feoktistov and Biaggioni, 1997; Zocchi et al., 1996), and erythro-9-(2-hydroxy-3-nonyl) (EHNA, an inhibitor of adenosine deaminase [ADA]) were purchased from Sigma-Aldrich and were dissolved as a 1 mM stock solution in DMSO and diluted 1/10000 in culture medium before use. Toll-like receptor ligands lipopolysaccharide (LPS) and Pam3csk4 (Pam3) and ADA polyclonal antibody were purchased from Invivogen (San Diego, CA, USA).

### 2.2 Immunization and EAU induction

EAU was induced in B6 mice by subcutaneous injection of 200 μl of emulsion containing 200 μg of human interphotoreceptor retinoid-binding protein (IRBP)_1-20_ (Sigma-Aldrich) in complete Freund's adjuvant (CFA; Difco, Detroit, MI, USA) at six spots at the tail base and on the flank and intraperitoneal injection with 300 ng of pertussis toxin.

### 2.3 T cell preparations

αβ T cells were purified from B6 mice immunized with IRBP_1-20_, as described previously (Cui et al., 2009; Liang et al., 2013b; Nian et al., 2010), while γδ T cells were purified from immunized and control (naïve) B6 mice. Nylon wool-enriched splenic T cells from naive or immunized mice were incubated sequentially for 10 min at 4 °C with FITC-conjugated anti-mouse γδ TCR or αβ TCR Abs and 15 min at 4 °C with anti-FITC Microbeads (Miltenyi Biotec GmbH, Bergisch Gladbach, Germany), then separated into bound and non-bound fractions on an autoMACS™ separator column (Miltenyi Biotec GmbH). The purity of the isolated cells, determined by flow cytometric analysis using PE-conjugated Abs against αβ or γδ T cells, was >95 %.

### 2.4 Prepare γδ T cells

Non-activated and activated γδ T cells were separated from either naïve B6 mice or IRBP_1-20_-immunized B6 mice (Liang et al., 2013b) (Liang et al., 2016a), respectively, by positive selection using a combination of FITC-conjugated anti-TCR-δ antibody and anti-FITC antibody-coated Microbeads, followed by separation using an auto-MACS.

### 2.5 Measurement of Th1 and Th17 responses

αβ T cells (1.8 × 10^6^) were collected from IRBP_1-20_-immunized B6 mice on day 13 post-immunization, based on previous tests showing that highest T cell responses are detected on days 13–15 post immunization. To obtain a sufficient number of cells, we routinely pool the cells obtained from all six mice in the same group, before the T cells are further enriched. The cells were co-cultured for 48 h with irradiated spleen cells (1.5 × 10^6^/well) as antigen presenting cells (APCs) and IRBP_1-20_ (10 μg/ml) in a 24-well plate under either Th1 (culture medium supplemented with 10 ng/ml of IL-12) or Th17 polarized conditions (culture medium supplemented with 10 ng/ml of IL-23) (Liang et al., 2013b, 2014a). Cytokine (IFN-γ and IL-17) levels in the serum and 48 h of culture supernatants were measured by ELISA (R&D Systems). The percentage of IFN-γ^+^ and IL-17^+^ T cells among the responder T cells was determined by intracellular staining 5 days after in vitro stimulation, followed by FACS analysis, as described previously (Liang et al., 2014a).

### 2.6 Generation of bone marrow dendritic cells

Bone marrow dendritic cells (BMDCs) were generated by incubating bone marrow cells for 5 days in the presence of 10 ng/ml of recombinant murine GM-CSF and IL-4 (R&D Systems), as described previously (Inaba et al., 1992). Cytokine (IL-1β, IL-6, L-12 and IL-23) levels in the culture medium were measured by ELISA after BMDCs were treated with AR agonists. To determine antigen-presenting function, BMDCs were incubated in a 24-well plate with responder T cells isolated from immunized B6 mice under Th1- or Th17-polarizing conditions. Forty-eight hours after stimulation, IFN-γ and IL-17 in the culture medium were measured by ELISA. The percentage of IFN-γ^+^ and IL-17^+^ T cells among the responder T cells was determined by intracellular staining after 5 days of culture as described above.

### 2.7 Intracellular cytokine flow cytometry

Unfractionated or purified CD3^+^ T cells isolated from immunized mice were stimulated in vitro with 50 ng/ml of PMA, 1 μg/ml of ionomycin and 1 μg/ml of brefeldin A (Sigma-Aldrich, St. Louis, MO) for 4 h, then washed, fixed, permeabilized overnight with Cytofix/Cytoperm buffer (eBioscience, San Diego, CA). The cells were then intracellularly stained with antibodies against IFN-γ and IL-17 and analyzed on a FACScalibur flow cytometer.

### 2.8 Carboxyfluorescein succinimidyl ester (CFSE) assay

Purified αβ T cells from IRBP1-20-immunized B6 mice were stained with carboxyfluorescein succinimidyl ester (CFSE, Sigma-Aldrich) as described previously [34]. Briefly, the cells were washed and suspended as 50 × 10^6^ cells/ml in serum-free RPMI 1640 medium (Corning Cellgro, VA) and incubated at 37 °C for 10 min with gentle shaking with a final concentration of 5 μM CFSE. The cells were then washed twice with RPMI 1640 medium containing 10 % fetal calf serum (Atlantic Inc. Santa Fe, CA, USA; complete medium), suspended in complete medium, stimulated with immunizing peptide in the presence of irradiated syngeneic spleen cells as antigen-presenting cells (APCs), and analyzed by flow cytometry.

### 2.9 ELISA measurement of cytokine

Purified αβ T cells (3 × 10^4^ cells/well; 200 μl) from the draining lymph nodes and spleens of IRBP_1-20_-immunized B6 mice were cultured in complete medium at 37 °C for 48 h in 96-well microtiter plates with irradiated syngeneic spleen APCs (1 × 10^5^) in the presence of 10 μg/ml of IRBP_1-20_. A fraction of the culture supernatant was then assayed for IL-17 and IFN-γ, using ELISA kits (R & D Systems).

### 2.10 Statistical analysis

The results in the figures are representative of one experiment, which was repeated 3–5 times. Data were analyzed using a paired *t*-test. A P value < 0.05 was considered a statistically significant difference and was marked with ** when P < 0.01.

## 3 Results

### 3.1 Adenosine preferentially inhibits Th1 but not Th17 responses

**Fig. 1:**
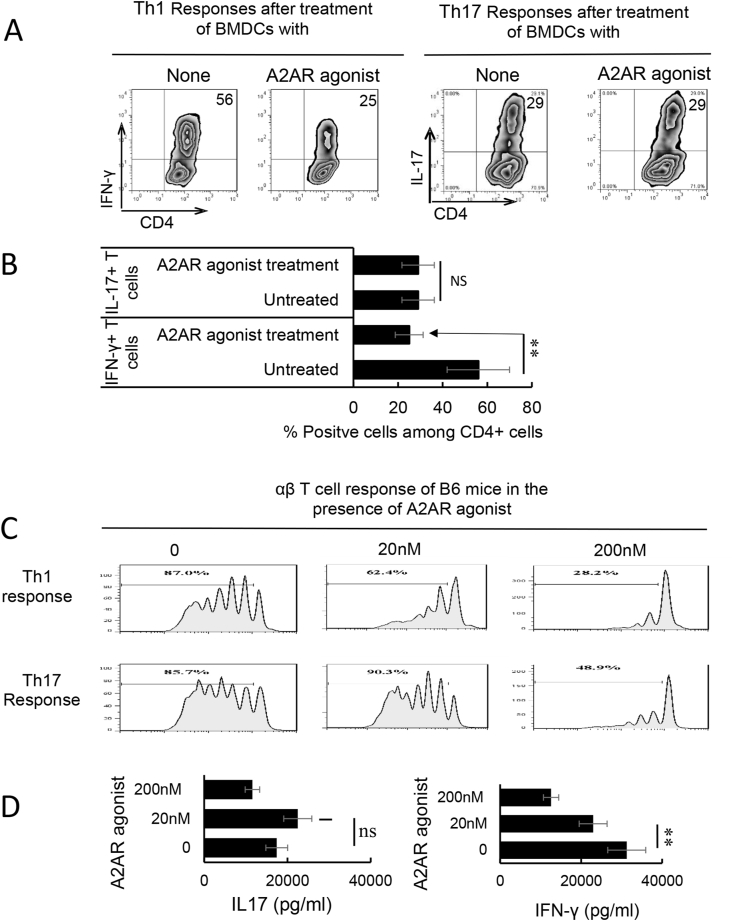
The effect of adenosine receptor agonist on Th17 responses differed from its effect on Th1 responses. A&B) B6 mice were immunized with interphotoreceptor retinoid-binding protein (IRBP)_1-20_/complete Freund's adjuvant (CFA). Thirteen days after immunization, CD3^+^ cells were separated from spleen and draining lymph nodes cells of immunized mice using a MACS column. They were stimulated with the immunizing peptide (IRBP_1-20_) and APCs, in the absence or presence of an A2AR agonist (CGS21680, 250 nM), under Th17 (right panels) or Th1 (left panels) polarized conditions. The numbers of αβTCR^+^ IL-17^+^ cells were assessed after a 5-day in vitro stimulation by FACS analysis. Data summarized for 4 separate experiments are plotted in (B). Data were analyzed using a paired *t*-test. **p < 0.01; ns, not significant, n = 6 in each group. C) Carboxyfluorescein succinimidyl ester assay for assessing dose-dependent effect (0–200 nM) of A2AR agonist (CGS21680) on Th1 and Th17 response. MACS column-separated CD3^+^ cells of immunized B6 mice were stimulated with the immunizing peptide (IRBP_1-20_) and antigen presenting cells, under Th17 or Th1 polarized conditions, in the presence of indicated doses of CGS21680. The numbers of activated T cells were assessed by FACS analysis after a 5-day in vitro stimulation. The results shown are representative of those from five experiments. D). Calculated inhibition of Th1 and Th17 response by graded doses of CGS21680. The graphs are showing SEM. Data were analyzed using a paired *t*-test. **p < 0.01; ns, not significant, n = 6 in each group.

To determine the adenosine effect on Th1 and Th17 responses in EAU, CD3^+^ responder T cells were harvested 13 days post immunization from the spleens and draining lymph nodes of B6 mice immunized with a uveitogenic antigen (IRBP_1-20_). The responder T cells were stimulated in vitro with the immunizing peptide and APCs (irradiated spleen cells), in the absence or presence of a selective A2AR agonist (CGS21680), under culture conditions that favor Th17 or Th1 autoreactive T cell expansion (medium containing 10 ng/ml, respectively, IL-23 or IL-12) (Liang et al., 2013b; Zuo et al., 2012). Th1 and Th17 responses specific for the immunizing antigen were estimated by assessing responding IFN-γ^+^ and IL-17^+^ T cells after intracellular staining with Fluorescence-labeled anti-IFN-γ or anti–IL-17 antibodies (Fig. 1A&B). The results showed that the number of IFN-γ^+^ cells in response to CGS21680 decreased significantly, whereas the number of IL-17^+^ T cells remained unchanged. We have previously shown that γδ T cell was a major contributor to the regulation of Th17 responses. To determine whether adenosine would have similar effect on Th1 and Th17 responses in the absence of γδ T cells, which play a major role in Th17 responses (Nian et al., 2011; Rajan et al., 2000; Spahn et al., 1999), we prepared responder CD3^+^ T cell from immunized TCR-δ^−/−^ mice and assessed T cell activation in the presence of varying doses of A2AR agonist using a CFSE assay (Fig. 1C), in which the responder cells were pre-labeled with CFSE before stimulation under polarizing conditions. The results show that in the absence of γδ T cells both Th1 and Th17 responses are inhibited by A2AR agonist. However, the Th1 responses were readily inhibited by a very low dose (20 nM) of the A2AR agonist that is inhibitory for Th1 response; but the Th17 responses remained minimally affected unless a very high dose (>200 nM) of the A2AR agonist was tested. Measurement of cytokine production of the responder T cells showed that IFN-γ production was inhibited by a very low dose (20 nM) of A2AR agonist while IL17 production was only inhibited by doses of A2AR agonist that were 10 times higher (Fig. 1D).

### 3.2 γδ T cells offset an inhibitory effect of A2AR agonist on Th17 responses

**Fig. 2:**
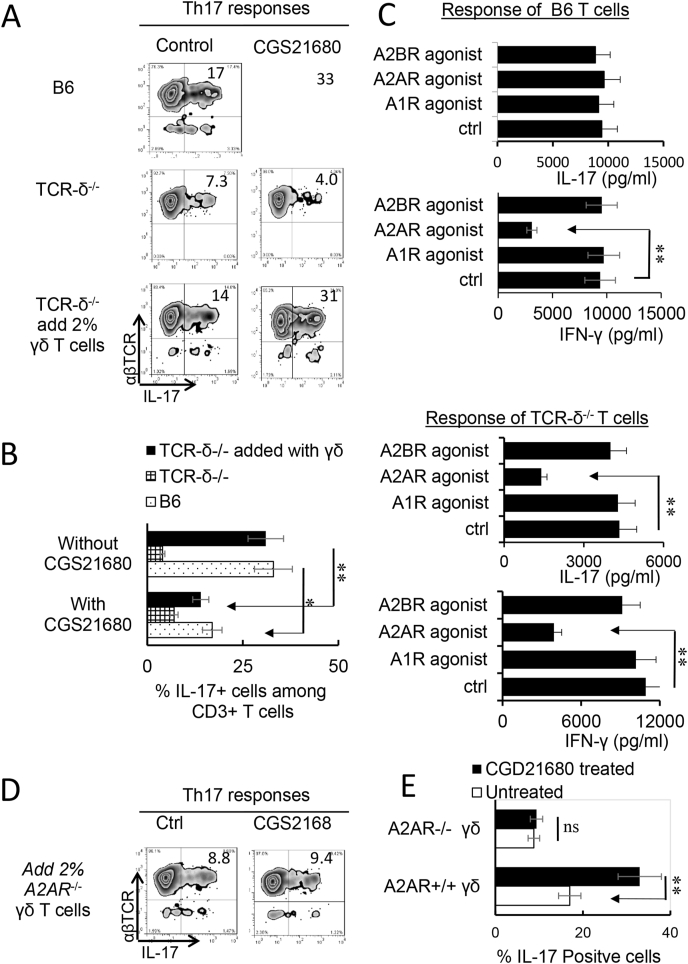
γδ T cell offsets the inhibitory effect of A2AR agonist on Th17 responses. A) Intracellular staining of IL-17^+^ T cells among the responder T cells. Responder T cells were separated from either immunized B6 (top panels) TCR-δ^−/−^ mice without (mid panels) or with (lower panels) 2 % supplemented γδ T cells. After stimulation with the immunizing peptide interphotoreceptor retinoid-binding protein (IRBP)_1-20_ and antigen presenting cells (APCs), under Th17 polarized conditions. The numbers of αβTCR^+^ IL-17^+^ and αβTCR^+^ IFN-γ^+^ cells were assessed by FACS analysis after a 5-day in vitro stimulation. The results shown are representative of those from five experiments. B). Summary data for all 5 replicates of Fig. 2A. Data were analyzed using a paired *t*-test. **p < 0.01, n = 6 in each group. C). ELISA test assesses IL-17 (upper panels) and IFN-γ production (upper two panels) by B6 (left panels) and TCR-δ^−/−^ responder T cells (lower two panels) under effect of agonists for specific adenosine receptors A1R (CCPA, 50 nM), A2AR (CGS 21680, 250 nM), A2BR (BAY60-6538, 100 nM), and vehicle control. The graphs show SEM. Data were analyzed using a paired *t*-test. **p < 0.01, n = 6 in each group. D). Th17 responses of TCR-δ^−/−^ responder T cells were not enhanced by A2AR^−/-^ γδ T cells. Responder T cells of TCR-δ^−/−^ mice were supplemented by 2 % A2AR^−/-^ γδ T cells (controls of adding A2AR^+/+^ γδ T cells were shown in Fig. 2A), before stimulation with IRBP_1-20_ and APCs, under Th17 polarized conditions. The numbers of αβTCR^+^IL-17^+^ cells were assessed by FACS analysis after a 5-day in vitro stimulation. E). A summary data for all 3 replicates of Fig. 2D are shown. Data were analyzed using a paired *t*-test. **p < 0.01; ns, not significant, n = 6 in each group.

Previous studies showed that γδ T cells are important enhancers of Th17 responses (Nian et al., 2011; Rajan et al., 2000; Spahn et al., 1999). To determine the mechanism by which A2AR agonist is more inhibitory for Th1 responses than Th17 response, we compared Th17 responses in the presence or absence of γδ T cells. The CD3^+^ T cells containing γδ T cells were purified from immunized B6 mice. Those not containing γδ T cells were prepared from immunized TCR-δ^−/−^ mice. The T cells were stimulated in vitro with the immunizing peptide and APCs, and the Th1 and Th17 responses were determined by the number of αβTCR^+^IFN-γ^+^ and αβTCR^+^IL-17^+^ T cells among responder T cells and the amount of IFN-γ and IL-17 produced in culture supernatants by ELISA. The results in Fig. 2A showed that the generation of αβTCR^+^IL-17^+^ cells from wild-type (WT) B6 responders (Fig.2A, top panels) was enhanced by the A2AR agonist CGS21680; but cells from TCR-δ^−/−^ responders (Fig. 2A, second panels) were not enhanced. Moreover, if 2 % of γδ T cells were added to TCR-δ^−/−^ responder T cells before in vitro stimulation, their responses were also enhanced (Fig. 2A third panels) suggesting that γδ T cells in responder T cells counteracted any inhibitory effect of adenosine, leading to greater Th17 responses. Measurement of Th1 responses under Th1-polarized conditions, however, showed γδ T cells are less effective in Th1 responses (data not shown). Cytokine production tests after in vitro stimulation showed that IL-17 production was inhibited by the A2AR agonist CGS21680 in TCR-δ^−/−^ CD3^+^ responders (third panel of Fig. 2C) but not in the responder T cells of B6 mouse (first panel of Fig. 2C), because the presence of γδ T cells among the B6 responders offset the inhibitor effect of CGS21680 (Cui et al., 2009; Liang et al., 2013b, 2016a; Nian et al., 2010). The IFN-γ production of both responders was inhibited regardless of whether γδ T cells were absent or present, indicating that Th1 inhibition by CGS21680 was not γδ T cell dependent. Studies comparing the effect of agonists specific for the ARs A1R, A2AR and A2BR showed that agonists for A2BR and A1R ARs were also ineffective in inhibiting IL-17 production (Fig. 2C). Since A2ARs are not strictly expressed on γδ T cells, we also compared the adenosine effect on Th17 responses of TCR-δ^−/−^ responder T cells supplemented with A2AR^+/+^ (from B6 mice) or A2AR^−/-^ γδ T cells (from A2AR^−/-^ mice). The results showed that adenosine was unable to enhance the Th17 responses supplemented with A2AR^−/-^ γδ T cells (Fig. 2D&E), suggesting that binding of A2ARs to γδ T cells crucially involved adenosine-enhanced Th17 responses.

### 3.3 Adenosine augmented the Th17, but not Th1-stimulating effect of BMDCs triggered by a TLR ligand

**Fig. 3:**
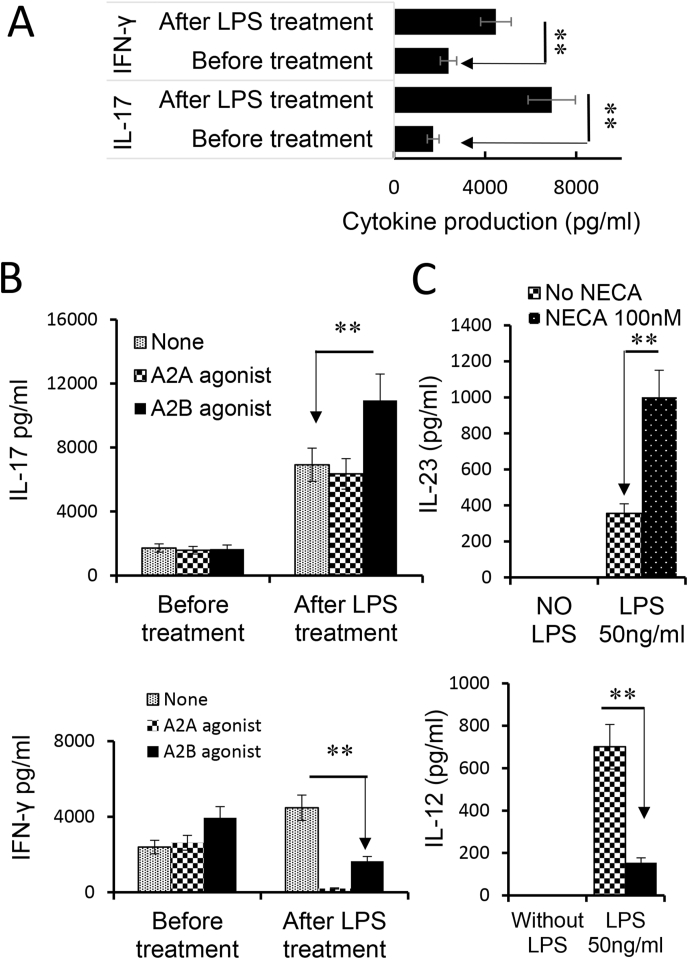
Adenosine augmented the Th17-, but not Th1-, stimulating effect of BMDCs triggered by TLR ligand. A) LPS treated BMDCs acquired an increased stimulating effect on Th1 and Th17 responses. Responder T cells were isolated from immunized B6 mice (n = 6). They were stimulated with the immunizing peptide interphotoreceptor retinoid-binding protein (IRBP)_1-20_ and bone marrow dendritic cells (BMDCs), under Th1 (upper panels) or Th17 (lower panels) polarized conditions. Cytokines in the supernatants were assessed by ELISA 48hr after stimulation. B). Dissociated Th1 and Th17 stimulating effect of BMDCs after dual treatment with lipopolysaccharide (LPS) and adenosine. BMDCs were treated with A2AR agonist (250 nM) or A2BR agonist (100 nM) before (left panels) or after (right panels) LPS treatment. After co-culture with responder T cells, IFN-γ and IL-17 amounts in culture supernatants were determined by ELISA. The results show that after LPS treatment, A2BR agonist treatment augmented BMDCs' Th17-stimulating effect, whereas both A2AR and A2BR agonists decreased BMDCs' Th1-stimulating effect. C). IL-12 and IL-23 production by BMDCs after treatment with LPS, with or without AR agonist. BMDCs produce IL-12 and IL-23 only after treatment with LPS. When LPS treated BMDCs were further exposed to the AR agonist, the IL-12 production was declined, whereas the IL-23 production was significantly increased. The results shown are representative of those from five experiments. Data were analyzed using a paired *t*-test. **p < 0.01, n = 6 in each group.

Dendritic cells are the principal antigen-presenting (AP) cells for initiating immune responses. Previous studies showed that TLR ligands have a profound effect on DC differentiation and maturation (Fedele et al., 2005). Since the level of extracellular adenosine increases greatly during inflammation (Eltzschig et al., 2012; Ohta and Sitkovsky, 2001; Sitkovsky et al., 2004), we questioned whether adenosine and TLR ligands have counteractive or synergistic effects on DC function and Th1 and Th17 responses. To do so, we assessed GM-CSF-cultured BMDCs for an AP effect in Th1 and Th17 responses, before and after exposure to adenosine and/or TLR ligands. The responder T cells were co-cultured with the treated BMDCs at ratio of DC:T = 1:10 in the presence of immunizing antigen and the cytokine production of responder T cells was measured. After BMDCs were treated with LPS only, both IFN-γ and IL-17 production were increased (Fig. 3A). Unexpectedly, when BMDCs were treated with LPS and A2BR agonists IFN-γ and IL-17 production changed in opposite directions; IL-17 increased whereas IFN-γ declined (Fig. 3B). Thus, the Th1 and Th17-stimulating effects of BMDCs were dissociated under a dual effect of TLR ligand and adenosine, tipping the Th1 and Th17 balance towards the latter. We then investigated whether the higher Th17-promoting effect of adenosine was associated with altered cytokine production by BMDCs after exposure to LPS and/or adenosine. Our results showed that BMDCs did not produce the cytokines tested before the LPS exposure (not shown); treatment with either LPS (TLR4 ligand) or PAM3 (TLR2 ligand) stimulated a low production of all tested cytokines, including IL-12, IL-23, L-1β and IL-6. After LPS and adenosine stimulation, IL-12 production declined and IL-23 production further increased, indicating the dissociated Th1 and Th17 responses can be partly attributed to altered cytokine production of BMDCs. Given that IL-23 (Cua et al., 2003; Peng et al., 2007) and IL-1β (Bettelli et al., 2007; Korn et al., 2007; Veldhoen et al., 2006) have a strong Th17-promoting effect, changes in patterns and amounts of cytokine production by BMDCs after adenosine presumably contributed to enhanced Th17 T cell response (Fig. 3C).

### 3.4 Adenosine augmented cytokine-mediated γδ T cell activation

**Fig. 4:**
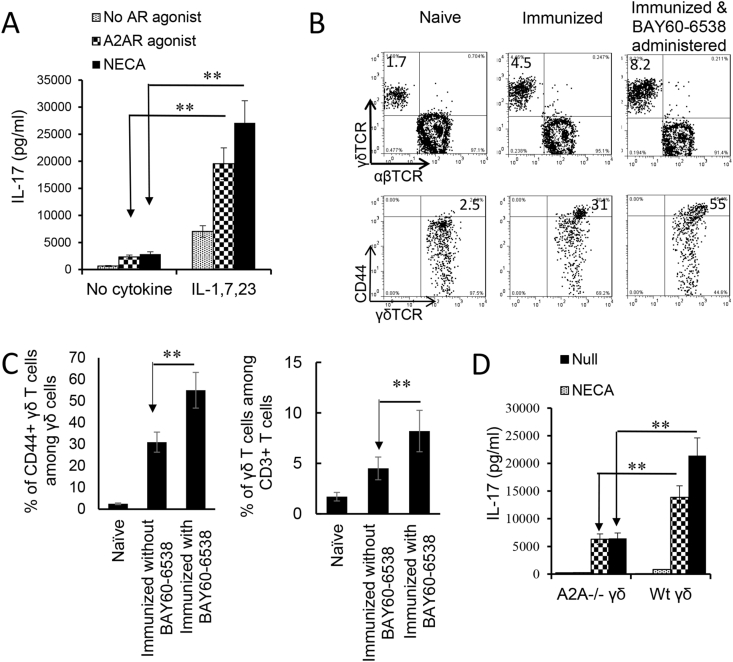
Adenosine analogue 5′-N-ethylcarboxamidoadenosine (NECA) augmented cytokine-mediated γδ T cell activation. A). IL-17 production by γδ T cells stimulated by cytokines is augmented by the analogue NECA. MACS purified γδ T cells were isolated from immunized B6 mice. In a 96-well plate, 2 × 10^5^/well γδ T cells were exposed to cytokines (IL-1β+7 + 23), in the absence or presence of the non-selective receptor ligand NECA or A2AR specific (250 nM) agonists. IL-17 in the cultured cell supernatants were assessed by ELISA. The results shown are summarized from four separate experiments. Data were analyzed using a paired *t*-test. **p < 0.01, n = 6 in each group. B). In immunized B6 mice administered with A2BR agonist (Bay60-6538), the number of total γδ T cells among CD3^+^ T cells (upper panels) and the number of activated (CD44^+^) γδ T cells (lower panels) are increased. B6 mice (n = 6) were immunized with interphotoreceptor binding protein (IRBP)_1-20_/complete Freund's adjuvant (CFA) with or without an injection of A2BR agonist (Bay60-6538, 1 mg/ml)). Thirteen days post-immunization CD3^+^ cells isolated were assessed for both abundance and activation status of γδ T cells. The CD3^+^ cells were gated for assessing total γδ T cells (upper panels) and γδTCR^+^ T cells were gated (lower panels) when CD44^+^ γδ T cells were determined. The results shown in Fig. 4B are from a single experiment. Summarized data of 4 separate experiments are shown in Fig. 3C. C). Data summarized for 4 separate experiments of Fig. 4B are plotted in Fig. 4C. Data were analyzed using a paired *t*-test. **, p < 0.01, n = 6 in each group. D). γδ activation is compromised if A2ARs on γδ T cells are disabled. γδ T cells isolated from B6 (A2AR^+/+^) or A2AR^−/-^ mouse (A2AR^−/-^) were compared for response to cytokines (IL-1β+7 + 23) and/or NECA. γδ activation is assessed by measurement of IL-17 production. Results show that adenosine enhances A2AR^+/+^ but not A2AR^−/-^ γδ activation. Data were analyzed using a paired *t*-test. **p < 0.01, n = 4 in each group.

Given our previous findings that γδ T cell activation was a major contributor to the regulation of Th17 responses, we questioned whether the enhancing effect of adenosine on Th17 responses was due to augmented γδ T cell activation. As we have previously reported, purified γδ T cells can be activated by a number of proinflammatory cytokines and that a mixture of IL-1β, IL-7, and IL-23 has a strong stimulatory effect (Liang et al., 2013b). We used this combination and tested the activation of γδ T cells by cytokines and in the absence or presence of adenosine. Responder γδ T cells were prepared from immunized B6 mice using MACS sorter. Fig. 4A shows that cytokines IL-1β, IL-7, and IL-23 were able to activate IL-17 production of γδ T cells; furthermore, a combination of adenosine analogue NECA and the cytokine mixture greatly augmented IL-17 production by γδ activation, even though neither NECA nor A2AR agonist itself appreciably stimulated γδ T cells. A similar synergistic effect was seen when γδ T cells were exposed to a combination of a TLR ligand and NECA (not shown). Assessment of the in vivo effect of adenosine on γδ T cells showed that B6 mice that received an A2BR agonist (BAY60-6538) injection after immunization had greater numbers of γδ T cells, among which the CD44^high^ γδTCR ^+^ cells were more abundant (Fig. 4B&C). To further determine that adenosine is responsible for γδ T cell activation we also compared the activation of A2AR^+/+^ and A2AR^−/-^ γδ T cells by these cytokines. Our results showed that after stimulation with the same dose of cytokines, the activation of A2AR^−/-^ γδ T cells was significantly lower than that of A2AR^+/+^ γδ T cells because the AR A2AR^−/-^ on γδ T cells was disabled (Fig. 4D).

### 3.5 Adenosine augmented the TLR ligand activation of γδ T cells by BMDCs

**Fig. 5:**
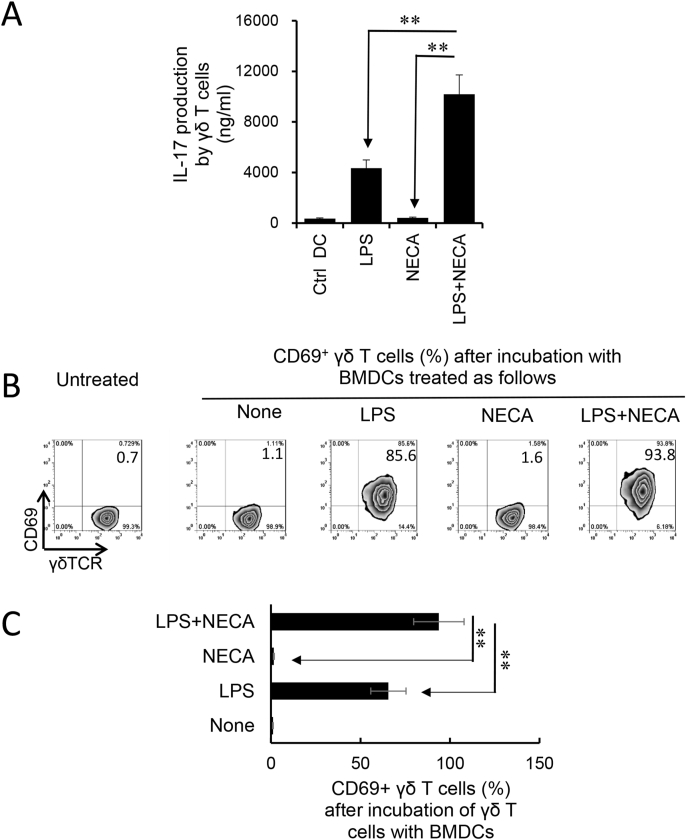
Synergistic effect of 5′-N-ethylcarboxamidoadenosine (NECA) and Toll-like receptor (TLR) ligand in bone marrow dendritic cells' (BMDCs') γδ-stimulating activity. A). BMDC-stimulated γδ T cells produced increased amounts of IL-17 if the BMDCs were pre-treated with lipopolysaccharide (LPS) and/or NECA. Data summarized for 4 separate experiments are shown. Data were analyzed using a paired *t*-test. **p < 0.01, n = 4 in each group. B&C). BMDC-stimulated γδ T cells expressed increased levels of CD69 after treated with LPS or LPS&NECA. The gated γδTCR^+^ T cells were further analyzed. One representative experiment from four separate ones is shown. **p < 0.01. Summarized data of three separate experiments are shown in Fig. 5C.

An alternative pathway of γδ T cell activation is stimulation by DCs. To determine whether BMDCs exposed to adenosine acquired an increased ability to stimulate γδ T cells, GM-CSF cultured BMDCs were co-incubated with MACS-sorted γδ T cells, after treatment with LPS and/or NECA, at a ratio of T:DC = 10:1 for two days. The activation of γδ T cells was assessed by measuring IL-17 production and the numbers of CD69^+^ γδ T cells. The results showed that only the only LPS-treated BMDCs could stimulate γδ T cells to produce IL-17, and BMDCs treated with LPS plus NECA acquired a greater stimulating effect (Fig. 5A). However, BMDCs treated with NECA alone were not stimulatory, indicating that the effect of adenosine on BMDCs is indirect and needed to be synergized with cytokines. Expression of CD69 – a cell surface marker identifying activated T cells - showed that only activated γδ T cells stimulated by LPS-treated BMDCs could augment γδ activation leading to augmented Th17 responses; furthermore, treatment of BMDCs with LPS plus NECA further augmented the stimulating effect of adenosine (Fig. 5B).

### 3.6 Inhibition of ADA by an ADA inhibitor augmented the IL17 responses

**Fig. 6:**
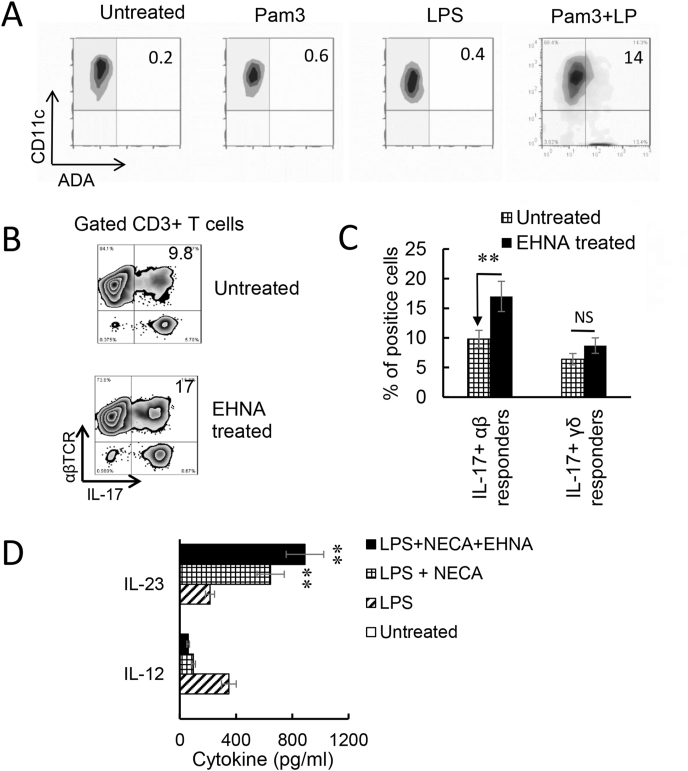
Adenosine deaminase (ADA) inhibitor erythro-9-(2-hydroxy-3-nonyl) adenine (EHNA) inhibited Th1 responses but enhanced the Th17 responses. A). Increased numbers of bone marrow dendritic cells (BMDCs) express ADA after Toll-like receptor (TLR) ligand stimulation. BMDCs were incubated with lipopolysaccharide (LPS) (100 ng/ml), Pam3 (2 μg/ml), or LPS + Pam3 for 48h, before they were stained with a polyclonal anti-ADA antibody. B). Prior treatment of BMDCs with EHNA augmented Th17 responses. Responder CD3^+^ T cells isolated from immunized B6 mice (n = 6) were stimulated, under Th17 polarized conditions, with BMDCs with (upper panels) or without (lower panels) a prior treatment with EHNA (10 μM). IL-17 Expression of CD3^+^ responder T cells were evaluated by FACS analysis after intracellular stain. Results show that inhibition of ADA by EHNA on BMDCs enhanced Th17 responses. One representative stain assay is shown. C). Data summarized for 3 separate experiments are shown. Data were analyzed using a paired *t*-test. **p < 0.01; ns, not significant, n = 4 in each group. D) BMDCs produced increased amounts of IL-23 after treatment with EHNA. BMDCs were treated with LPS (100 ng/ml), LPS plus 5′-N- ethylcarboxamidoadenosine (NECA) (100 nM), or LPS, NECA and EHNA (10 μM) as indicated. IL-12 and IL-23 amounts in supernatants were tested 48 h after stimulation. Data were analyzed using a paired *t*-test. **p < 0.01, n = 4 in each group.

Endogenously produced adenosine is degraded by ADA. We observed that Toll ligand activated BMDCs expressed increased amounts of ADA (Fig. 6A). To determine whether Th1 and Th17 responses would be affected if ADA function is deactivated, we determined the AP function of BMDCs with or without prior treatment with EHNA ─ a reversible inhibitor of ADA (North and Cohen, 1978; Ullman et al., 1976). The results showed that inhibition of ADA by EHNA enhanced both the Th17 and γδ T cell responses (Fig. 6B). Measurement of cytokine production of BMDCs showed that untreated BMDCs produced neither IL-23 nor IL-12, but the production of these cytokines was induced by LPS. Adenosine analogue NECA inhibited Il-12 production but enhanced IL-23 production. If the BDMCs were simultaneously treated with EHNA, the IL23 production of BMDCs further increased. These results supported the prediction that regulation of endogenously generated adenosine by ADA crucially controls adenosine levels, and thus controls Th17 responses; when ADA is disabled, adenosine will accumulate and Th17 responses will be enhanced.

## 4 Discussion

During stress and tissue injury, ATP is released from the intracellular compartment into the extracellular space, where it is degraded to adenosine through a cascade of enzymatic reactions. Elevated amounts of adenosine are found in ischemia, inflammation and trauma (Fredholm et al., 2001; Haskó et al., 2008; Idzko et al., 2014; Linden, 2001). Degradation of ATP to adenosine involves ectonucleotidases including CD39 (nucleoside triphosphate diphosphorylase [NTPDase]) and CD73 (5′-ectonucleotidase [Ecto5′NTase]) (Haskó et al., 2009; Yegutkin, 2008). Produced adenosine is degraded by ADA (North and Cohen, 1978; Ullman et al., 1976). Adenosine is an important regulatory molecule since it modulates a wide range of physiological functions (Fredholm et al., 2011) including the immune response (Fredholm et al., 2011; Haskó et al., 2008; Jacobson and Gao, 2006; Sauer et al., 2012) by acting on many types of immune cells, including T cells (Jin et al., 2010; Lappas et al., 2005), macrophages/DCs (Naganuma et al., 2006; Panther et al., 2001), NK cells (Hoskin et al., 2008), neutrophils (Fredholm et al., 2001), platelets (Varani et al., 1996), and regulatory T cells (Ehrentraut et al., 2012; Naganuma et al., 2006; Zarek et al., 2008).

Four types of ARs have been defined, designated A1R, A2AR, A2BR, and A3R (Haskó et al., 2008; Ohta and Sitkovsky, 2001). The major functional receptor on T cells is A2AR (Ohta and Sitkovsky, 2001; Sitkovsky and Ohta, 2005). Previous studies have demonstrated that adenosine has a direct inhibitory effect on αβ T cells and macrophages/DCs (Erdmann et al., 2005; Huang et al., 1997; Naganuma et al., 2006; Ohta et al., 2006; Panther et al., 2001; Schnurr et al., 2005; Sevigny et al., 2007). Treatment with adenosine reduced Th1 responses (Haskó et al., 2000; Panther et al., 2003), and activation of A2AR on T cells inhibited T-cell–mediated cytotoxicity, cytokine production (Ohta et al., 2009) and T-cell proliferation (Deaglio et al., 2007; Zhang et al., 2004) (Zarek et al., 2008). Regulatory T cells exert their suppressive action through the production of adenosine (Borsellino et al., 2007; Deaglio et al., 2007; Kobie et al., 2006). Adenosine inhibits IL-12 production by DCs via which Th1 responses are inhibited (Csóka et al., 2008). Indeed, A2AR^−/-^ mice developed more severe experimental autoimmune encephalomyelitis, and A2AR antagonism protects against experimental autoimmune encephalomyelitis (Mills et al., 2012); treatment with the A2AR agonist resulted in marked decreases in retinal inflammation in diabetic retinopathy (Ibrahim et al., 2011). Our recent study has tested the protective effect of ADA – an enzyme converting adenosine into functionally inactive molecules (Mandapathil et al., 2010), in mouse EAU (Liang et al., 2016b). We found that ADA treatment suppresses EAU only when administered to recipients 8–14 days postimmunization, or shortly before EAU expression. Also, treatment of recipients with the ADA inhibitor EHNA enhances EAU development (Liang et al., 2016b). A similar “timing effect” has been found when mice are treated with the nonselective AR agonist NECA, which inhibits autoimmune responses when used at an early stage after immunization, but inhibits the response when administration of the same amount of NECA at a late stage (at 8–14 days post immunization, or prior to the disease onset (manuscript in preparation), indicating that the role of AR ligation in autoimmune pathogenesis is affected by environmental factors. Further investigations are required for better success in application.

The extrapolation of adenosine as inhibitory was mostly obtained from studies of Th1 immune responses, since Th17 responses were discovered only recently. Given that both Th1 and Th17 pathogenic T cells contribute to the pathogenesis of autoimmune diseases (Bettelli et al., 2006; Cua et al., 2003; Dong, 2006; Kolls and Linden, 2004; Langrish et al., 2005), and since extracellular concentration of ATP and its metabolites is abundant at inflammatory sites (Haskó and Cronstein, 2004; Sitkovsky et al., 2004; Wilson et al., 2011), determination of whether adenosine has a similar effect on Th1 and Th17 pathogenic T cells is important. Determination of the mechanisms by which Th17 responses differed from Th1 autoreactive T cells in response to adenosine in EAU, a well-established mouse model of uveitis, showed that the effect of adenosine on Th17 responses is enhancing, while the predominant effect of adenosine in Th1 responses is anti-inflammatory (Panther et al., 2003) (Zarek et al., 2008). As a result, adenosine tips the Th1 and Th17 balance toward the latter. The opposite effect of adenosine on Th1 and Th17 responses could certainly offset therapeutic attempts to regulate Th1 pathogenic reactions. As such, clarification of the conflicting effect of adenosine on Th1 and Th17 responses is of major importance.

The promoting effect on Th17 responses of adenosine has been also previously observed (Wilson et al., 2011); however, comparative effects on Th1 and Th17 responses have not been. Here we show that the enhancing effects of adenosine on Th17 responses is accomplished via several pathways, of which γδ T cell activation is the most important. An important finding in this study is that adenosine inhibits the αβ T cell responses but enhances γδ T cell activation and that the enhancing effect of AR ligation on Th17 responses is modulated by γδ T cells. We have previously shown that activated γδ T cells acquire a greatly increased ability to enhance Th17 responses (Liang et al., 2016a; Nian et al., 2011). γδ T cells can be readily activated by a number of proinflammatory cytokines, in the absence of TCR ligation. For example, a mixture of IL-1, IL-7, and IL-23 has a strong stimulatory effect on γδ T cells (Liang et al., 2013a). When adenosine was added to the cytokine mixture, γδ T cell-activation was significantly enhanced, even though adenosine itself does not activate γδ T cells (Fig. 4). In the absence of γδ T cells, adenosine is inhibitory for both Th1 and Th17 responder T cells; however, when as few as 2 % γδ T cells were added to responder αβ T cells, adenosine inhibition of Th17 responses was abolished but the inhibitory effect on Th1 remained. Furthermore, the Th17 enhancing effect of γδ T cells was abolished when the effect of A2ARs on γδ T cells was disabled (Fig. 2), suggesting that the effect of adenosine on γδ T cells plays an important role in the enhanced responses of Th17. Adenosine promotes DC differentiation into a unique subset that strongly stimulates Th17, but not Th1, responses; in addition, it augments the γδ-stimulating activity of BMDCs, via which Th17 responses are further enhanced (Mills et al., 2012).

In the study of DCs’ we found that BMDCs have Th1-stimulating activity but very little Th17 stimulating capacity before adenosine treatment. After treatment with TLR ligands, both the Th1 and the Th17 stimulating effects on BMDCs were enhanced. Unexpectedly, when BMDCs were treated with both TLR ligand and adenosine, the Th1 and Th17-stimulating effects of BMDCs were dissociated; while the Th1-stimulating function declined, Th17 stimulation increased and tipped the Th1/Th17 balance towards the latter. Given that ATP may function as an endogenous TLR ligand (Matzinger, 2002; Ravichandran, 2010; Vitiello et al., 2012), it is likely that the balance of ATP and its degrading adenosine metabolites plays an important role in the T cell response. To investigate the function of ATP degradation and deactivation of adenosine by ADA enzyme we examined whether deactivation of ADA by a specific enzyme (EHNA) would result in excess adenosine and promote cascading Th17 responses. Our results demonstrated that ADA inhibition favors enhanced Th17 responses.

Alternative pathways may have been also involved in adenosine-induced enhancement of T cell responses. As we previously reported, activated γδ T cells express greatly increased amounts of high-affinity ARs (A2ARs) (Liang et al., 2014b, 2016a), leading to altered adenosine binding by various immune cells (Liang et al., 2014b). The preferential binding of adenosine by γδ T cells may lead to a re-distribution of adenosine among various immune cells, leading to diminished adenosine binding by αβ T cells, for example, which will also favor augmented αβ T cell responses (Liang et al., 2013b, 2014b, 2016a; Nian et al., 2011).

## 5 Conclusion

A better knowledge and understanding of the functional conversion of adenosine should facilitate adenosine-mediated immunotherapies. The cellular and molecular basis for enhancing and/or inhibiting the effects of ATP/adenosine remain to be further determined and the outcome of such studies should improve currently available therapies, including adenosine- and γδ T cell-based immunotherapies.

## Grant information

This work was supported by U.S. National Institutes of Health, 10.13039/100000002National Eye Institute Grants EY0022403 and EY018827 and by a grant from 10.13039/100001818Research to Prevent Blindness, NYC.

## Data sharing statement

The main data supporting the findings of this study are available within the paper.

## CRediT authorship contribution statement

**Deming Sun:** Formal analysis, Writing – original draft. **Minhee Ko:** Formal analysis, performed the experiments and analyzed data. DS and HK wrote the manuscript. All authors contributed to the article and approved the submitted version. **Hui Shao:** designed research. DS and. **Henry J. Kaplan:** Writing – original draft.

## Declaration of competing interest

The authors declare that they have no known competing financial interests or personal relationships that could have appeared to influence the work reported in this paper.
